# Analytical Validation of Two Point-of-Care Assays for Serum Amyloid A Measurements in Cats

**DOI:** 10.3390/ani11092518

**Published:** 2021-08-27

**Authors:** Damián Escribano, Alba Ortín Bustillo, Luis Pardo Marín, Andrea Navarro Rabasco, Pablo Ruiz Herrera, Jose J. Cerón, Asta Tvarijonaviciute

**Affiliations:** 1 Interdisciplinary Laboratory of Clinical Analysis, Interlab-UMU, Department of Animal Medicine & Surgery, Veterinary School, Campus Mare Nostrum, University of Murcia, 30100 Espinardo, Murcia, Spain; alba.ortinb@um.es (A.O.B.); lpm1@um.es (L.P.M.); andrea.navarror@um.es (A.N.R.); pablo.ruizh@um.es (P.R.H.); jjceron@um.es (J.J.C.); asta@um.es (A.T.); 2 Department of Animal Production, Veterinary School, Campus Mare Nostrum, University of Murcia, 30100 Espinardo, Murcia, Spain

**Keywords:** serum amyloid A, cat, inflammation, point-of-care-assays, analytical validation

## Abstract

**Simple Summary:**

Serum Amyloid A (SAA) is one of the most sensitive tests to detect inflammation in cats. In this study, two point-of-care assays for serum amyloid A measurements in cats were analytically evaluated. The two assays were accurate and showed significant correlations with an automated assay previously validated in cats, although one of them did not show an optimal precision. Both assays were able to detect higher SAA concentrations in cats with inflammatory diseases than in cats without inflammatory diseases. In conclusion, this manuscript provides data about the possible application of two point-of-care assays for the measurement of SAA concentration in cats.

**Abstract:**

Serum Amyloid A (SAA) is one of the most sensitive tests to detect inflammation in cats. In this study, two point-of-care assays for SAA measurements in cats (FUJI DRI-CHEM IMMUNO AU CARTRIDGE vf-SAA (method A), and CUBE-VET analyser (Method B), were analytically evaluated. Regarding the imprecision precision only the method A showed intra-assay and inter-assay CV < 10% at all concentrations. Both assays showed linearity with r close to 1 and the recovery were in the range of 81–112% for assay A and 85–125% for assay B and the limit of detection were 3.75 and 0.5 mg/dL for method A and B, respectively. A previously validated method for SAA quantification SAATIA; LZ-SAA (method C) was used as gold-standard to evaluate the accuracy of the assays. Significant correlations (*p* < 0.0001) were found between assays A and C (r = 0.94) and B and C (r = 0.91). In addition, an overlap performance test was made using serum samples from cats with non-inflammatory and cats with inflammatory. Both assays showed higher median SAA concentrations in cats with inflammatory diseases than in cats without inflammatory diseases (*p* < 0.0001). In conclusion, this manuscript provides data about the possible application of two point-of-care assays for the measurement of SAA concentration in cats.

## 1. Introduction

Acute phase proteins (APPs) are sensitive markers of inflammation. In recent years, in companion animals, the APPs have been included in routine biochemical profiles at a number of laboratories all over the world, giving rise to the possibility of their increasing use in routine clinical practice. APPs represent a very useful additional tool to the biochemical profile or haemogram, improving the clinical value of the laboratory data to detect and monitor inflammatory conditions. Some guidelines about accurate use and interpretation of APPs have been described, that can help practitioners to their use [1,2]. In these guidelines it is of importance to make an adequate analytical validation since validated assays should be always used for the measurement of APPs. Therefore, an analytical validation of any assay should be performed, including at least analytical precision and accuracy. In addition, an overlap performance test with healthy individuals and individuals with an inflammatory condition is recommended before the routine use of the assay in clinic [2].

In cats, serum amyloid A (SAA) is a major acute phase protein and it is increased in various conditions such as upper respiratory tract infection, pneumonia, pyometra, acute pancreatitis and feline infectious peritonitis [3]. In addition, *Candidatus Mycoplasma haemominutum* and *Mycoplasma haemofelis* increases SAA in cats [4]. In a report about SAA use in clinical setting, although increases values in SAA were found in cases of hyperthyroidism, diabetes mellitus, lymphoma, cholangitis, malignant mesothelioma or renal failure, the higher values of SAA in cats appear in acute pancreatitis and feline infectious peritonitis [5].

The use of point of care tests (POC) are of interest for rapid results that can be obtained in the clinic. In the case of SAA, a POC assay has been evaluated in horses [6]. However, in cats, although a turbidimetric immunoassay has been validated, to the author’s knowledge no analytical validation of POC tests have been made in this species [5,7].

The objective of the present study was to evaluate the analytical and overlap performance of two different point-of-care assays for in-house SAA measurements in cats: the FUJI DRI-CHEM IMMUNO AU CARTRIDGE vf-SAA (FUJI DRI-CHEM IMMUNO AU10V, FUJIFILM, Tokyo, Japan) and SAA VET test kit (CUBE-VET analyser, Eurolyser Diagnostica GmbH, Salzburg, Austria). A previously validated immunoturbidimetric assay SAATIA; LZ-SAA (Eiken Chemical Co., Ltd.; analyzer Olympus AU600, Tokyo, Japan) was used for comparative purposes [7]. We hypothesized that the SAA POC tests would have an acceptable analytical and overlap performance in serum. It is expected that this validation study would increase the knowledge about the analytical performance of POC assays for SAA measurement in cats and allow a wider use of this APP in clinical practice.

## 2. Materials and Methods

The study was performed using serum samples from 130 cats, 79 for the correlation study and 51 for the overlap performance study, that arrived between June 2019 and January 2020 to the Interdisciplinary Laboratory of Clinical Analysis (Interlab-UMU, University of Murcia, Spain) external analytical service and in which a biochemistry profile included SAA analysis was requested. Blood samples were not collected solely for this study but rather for either diagnostic purposes or for health assessments. Serum was collected from whole blood after clotting and centrifugation at 3500 rpm for 5 min at 4 °C. The obtained serum was stored in 1.5 mL vials at −80 °C until the time of analysis.

The assays used for the SAA measurement were:

- FUJI DRI-CHEM IMMUNO AU CARTRIDGE vf-SAA (Fujifilm^®^)

The FUJI DRI-CHEM IMMUNO AU CARTRIDGE vf-SAA test (method A) is based on Sandwich Immunoassay method. When a specimen is applied to a cartridge, the specimen and the dried fluorescence particle-labeled anti-SAA mouse monoclonal enclosed in the cartridge are mixed. SAA in the specimen reacts with a fluorescence particle-labeled antibody. The mixture then reacts continuously with Anti-SAA mouse monoclonal immobilized on the cartridge. The fluorescence particle-labeled antibody-SAA complex in the specimen binds to the solid-phase antibody. The fluorescence generated is directly proportional to the SAA concentration of the specimen. Considering the dead volume, 400 µL of the sample was used for the measurements. Measurement time is approximately 10 min.

- SAA VET test kit (Eurolyser Diagnostica GmbH)

It is a latex agglutination test (method B) based on optical measurement of the change in turbidity (photometric endpoint measurement at 546 nm wavelength) caused by the agglutination of the latex particles with an antibody against SAA. Required sample volume is 5 μL. The sample is pipetted into a cuvette containing a buffer and samples with concentrations higher than the upper limit of the measurement range should be diluted with physiological saline (0.9% NaCl). The cuvette is later filled with a solution containing anti-SAA antibodies. The reaction takes approximately 5 min.

- SAATIA; LZ-SAA (Eiken^®^ Chemical Co., Ltd.)

It is a human turbidimetric immunoassay (method C) designed for quantification of SAA in humans, but validated in cats [5,7]. It is based on turbidimetry and agglutination of latex particles. The commercial reagent (LZ-SAA) used a monoclonal antibody to SAA with latex particles adhered. In the presence of SAA in the sample, the latex particles undergo an agglutination reaction proportional to the concentration of the antigen in the sample. Required sample volume is 2 μL and the measurement time is approximately 10 min. Analyses were performed on an automated analyser (Olympus AU600, Olympus Diagnostica GmbH, Hamburg, Germany).

Imprecision was expressed as coefficient of variation (CV). Three pools (five samples to each pool) of saliva samples containing high, medium and low concentration of SAA were used. Intra-assay CV was determined by analysing the pools five times in a single analytical run whereas inter-assay CV by analysing them on six different days within 1 week. Coefficient of variation was calculated as percentage of the standard deviation of the replicates divided by the mean.

Accuracy of these assays was evaluated by linearity under dilution (1), a recovery study (2) and also by comparison with the Eiken immunoturbidimetric assay in a correlation study (3). (1) Linearity under dilution was determined by making serial dilutions of two pools of cat samples (four samples to each pool) with high concentration of SAA. Each dilution was assayed in triplicate. Results were then compared with those expected by linear regression analysis and a coefficient of determination (r) was calculated. (2) For the recovery experiment, the initial values of two samples were measured and after were analysed again after being mixed in different proportions (1:9; 5:5 and 9:1). Each sample was assayed in triplicate. Afterwards, observed and expected SAA concentrations were compared and percentages of recovery were calculated. (3) For the correlation study, a total of 79 serum samples with different SAA values were analysed.

The lower limits of detection (LLD) were be calculated by measuring 10 blank samples consisting in physiological saline (0.9% NaCl) solution.

For the overlap performance study, 25 cats with diseases associated with inflammation and 26 healthy cats were used. The cats with diseases associated with inflammation had feline infectious peritonitis (n = 12), pancreatitis (n = 10) and hyperthyroidism (n = 3). The healthy cats come to clinics for routine check-ups and did not have clinical signs at external examination. They also showed normal values of complete blood cell counts and biochemistry and were negative to the feline immunodeficiency virus antibody and feline leukaemia virus antigen tests.

Intra and inter-assay CVs, arithmetic means, SD and LLD were calculated in Microsoft Excel (Microsoft Corporation, Redmond, WA, USA, EE.UU). SAA results obtained from the overlap performance test were evaluated for normality of distribution using the Shapiro–Wilk test and showed a non-parametric distribution. A nonparametric Mann–Whitney test was performed to determine whether the values of SAA between the healthy cats and cats with inflammatory conditions showed statistically significant differences. Statistical analyses and the regression analysis were performed using GraphPad Prism version 8 (GraphPad Software, La Jolla, CA, USA). The significance level used in all the analyses was *p* < 0.05.

## 3. Results

### 3.1. Intra-Assay and Inter-Assay Reproducibility

Results from the precision study of all immunoassays are shown in Table 1. For method A and C all samples showed intra-assay CV < 10% at all concentrations. However, the method B had a CV > 10% at low concentrations (12.23%). In relation of inter-assay CV, the method A and C also showed values < 10% in all pool concentrations, whereas the method B showed CVs > 10% in all concentrations.

### 3.2. Accuracy

#### 3.2.1. Linearity under Dilution

The results found from the serial dilution of pools samples with high concentrations of SAA showed an r value close to 1 in all cases (A: r = 0.97; B: r = 0.97 and C: r = 0.97).

#### 3.2.2. Recovery Experiment

Recovery results are shown in Table 2. The recovery range of was between 81–112% for assay A; 85–125% for assay B and 81–117% for assay C.

#### 3.2.3. Correlation Study

**Figure 1:**
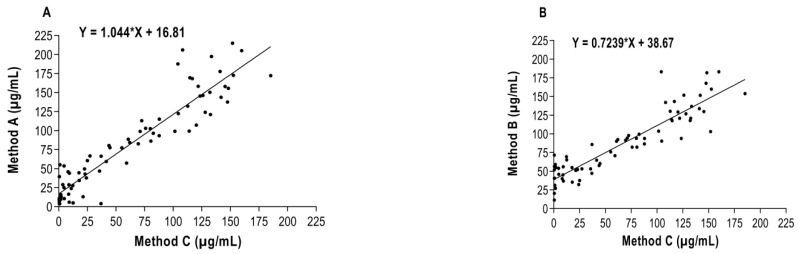
Correlation study of serum samples concentrations between: (**A**) Fujifilm (method A) and Eiken (method C) and (**B**) between Eurolyser Diagnostica (method B) and Eiken (method C).

The results of correlation study are shown in Figure 1. Significant correlations (*p* < 0.0001) were found between assays A and C (r = 0.94, n = 79) and B and C (r = 0.91, n = 71).

### 3.3. Lower Limit of Detection (LLD)

The LLD were 3.75 µg/mL, 10 µg/mL and 0.5 µg/mL for method A, B and C, respectively.

### 3.4. Overlap Performance

**Figure 2:**
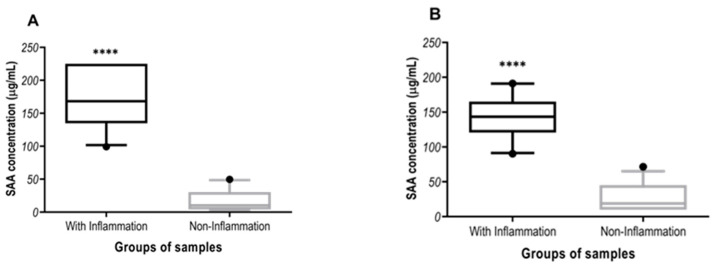

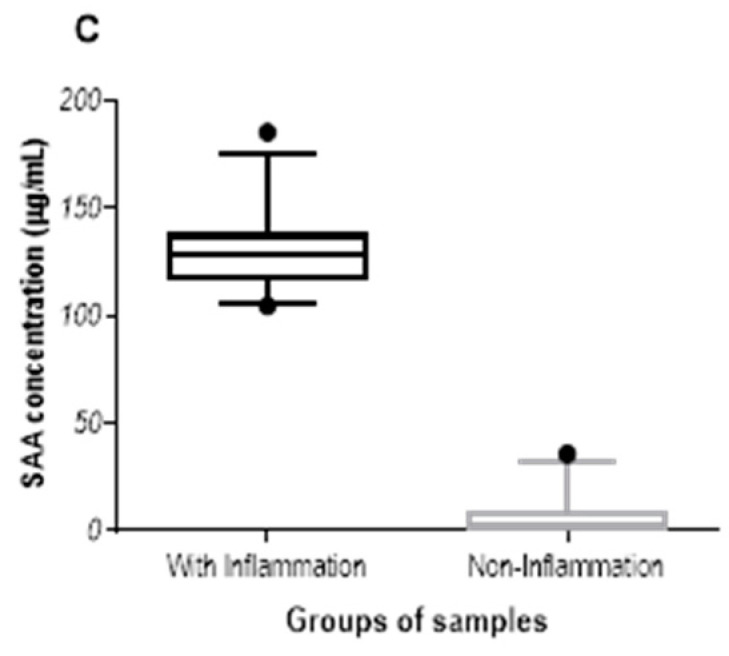
Serum Amyloid A concentrations in cats with different inflammatory diseases (n = 25) and cats from non-inflammatory diseases (n = 26) measured in Fuji (**A**), CUBE-VET (**B**) and Eiken (**C**) assay method. The plots show median (line within box), 25th and 75th percentiles (box) and 5th and 95th percentiles (whiskers). Asterisks indicated significant differences between groups: **** *p* < 0.0001.

In the overlap performance results, the three methods showed higher median SAA concentrations in cats with inflammatory diseases than in cats without inflammatory diseases (*p* < 0.0001, Figure 2). The SAA median concentrations in cats with inflammation were 168 µg/mL (99.3–225 µg/mL; 5th and 95th), 143 µg/mL (90.3–191 µg/mL; 5th and 95th) and 128 µg/mL (104–185 µg/mL; 5th and 95th) in method A, B and C, respectively. In case of cats without inflammatory diseases, the median concentration was of 10.2 µg/mL (3.75–49.6 µg/mL; 5th and 95th) in method A, of 18.9 µg/mL (10–71.6 µg/mL; 5th and 95th) in method B and of 1.75 µg/mL (0.2–35.7 µg/mL; 5th and 95th) in method C.

## 4. Discussion

In this report, two commercially available point of care assays for the quantification of SAA in cats were analytically validated. In addition, data about their ability to differentiate between inflammatory and non-inflammatory conditions were provided by an overlap performance study.

The results of the analytical validation showed that all assays were precise, with the exception of the assay B that showed an inter-assay imprecision higher than 20%, which is considered as the acceptable limit, with medium and low values [8]. There is a possibility that this imprecision would affect the differentiation between low and medium values. However, it would not decrease the ability to distinguish high values. All assays were linear; however, in our experience with high values of assays B and C, in some cases, they can lose their linearity due to the existence of a Hook effect. This could occur in situations in which in cat with high suspicion of inflammation based on the clinical exam and data from other analysis has SAA in normal values. Although the Hook effect of the point of care assays has not been validated in this study, clinicians should be aware of this possible Hook effect and a sample dilution would be recommended. The recoveries of all assays were in the acceptable range of 80–120% [9].

The two point of care assays were highly correlated with a previously validated automated assay and also all assays were able to discriminate between the high values of SAA, occurring in cats with inflammatory conditions, and the low values of SAA of healthy cats. Overall, our results would indicate that all assays can detect high values of SAA, and therefore they could help to raise the suspicion in the clinicians that an inflammatory process such as acute pancreatitis or feline infectious peritonitis is occurring [5]. In addition, increases in SAA have been detected in upper respiratory tract infections, pneumonia, pyometra or traumatic diseases among other clinical situations [5]. Further studies should be performed in order to establish specific reference ranges for each assay, since each assay gave different mean values in healthy cats in our study. It is important to point out that the SAA values should be evaluated in association with the data of clinical examination and other laboratory analysis, even involving other acute phase proteins, in order to provide an accurate interpretation. This study has a main limitation, which is the lack of use of pure feline SAA as reference material for the recovery assays. In addition, in the future further studies with a larger clinical validation involving more clinical cases with different inflammatory diseases of different intensity and degree of inflammation should be performed.

Regarding the lack of a larger clinical validation with a higher number of samples, the objective of our report was to perform an overlap performance study. This overlap performance is a phase of test validation whose purpose is to detect differences in analyte values between healthy and diseased individuals. According to previous guidelines in this phase, the analyte to study typically is measured in 20 to 30 healthy individuals and 20 to 30 individuals having a well-defined disease of interest [10]. Because this is meant to be a low-resource step, the material is often highly selected for having the clinical situation that it is wanted to be tested and, in this case, we included individuals with clear evidence of inflammation. Based on the results of this report in which no overlap in analyte concentrations was observed between healthy and inflamed groups, it can be stated that the diagnostic value of the tests evaluated in our study to detect inflammation is high [10].

Therefore, this report has completed the Phase 1 (Analytical performance) and Phase 2 (Overlap performance) of the assays’ validation and it has established the basis for future studies that could complete the Phase 3 (Clinical performance) in which the diagnostic capacity of a test (diagnostic sensitivity and specificity) in a population of clinical relevance is accurately defined [10].

## 5. Conclusions

This study provides data about the analytical validation and overlap performance of two point-of-care assays. These assays allow the measurement of SAA in in-house conditions and correlated with a validated automated assay previously validated in cats. Overall, these POC tests may be used to measure SAA concentrations in serum of cats, although some limitations of the assays (i.e., high imprecision at low and moderate values of assay B and the possibilities of hook effect) should be pointed out and taken in consideration for interpretation. It is expected that these data could contribute to a wider use of SAA assays in routine practice in cats for detecting and monitoring inflammation.

## Figures and Tables

**Table 1:** Intra-assay and inter-assay repeatability of the assays for detection of a high (>100 µg/mL), medium (≈50 µg/mL) and low (≈25 µg/mL) concentrations of feline serum amyloid A protein (SAA). The test assays used were Fujifilm (method A), Eurolyser Diagnostica (method B) and Eiken (method C).

			Intra Assay			Inter Assay	
SAA	Pool (n = 5)	X ^1^	SD ^2^	CV ^3^ (%)	X ^1^	SD ^2^	CV ^3^ (%)
Method A (μg/mL)	[High]	206.26	12.5	6.07	210.95	12.86	6.10
	[Medium]	64.21	0.59	0.93	62.66	2.30	3.68
	[Low]	25.15	0.53	2.12	28.83	0.84	3.16
Method B (μg/mL)	[High]	203.00	12.55	6.19	204.57	20.90	10.22
	[Medium]	60.40	2.00	3.33	59.17	13.10	22.15
	[Low]	27.68	15.86	12.23	41.75	10.38	24.88
Method C (μg/mL)	[High]	131.46	1.39	1.06	128.25	2.60	2.02
	[Medium]	60.44	0.45	0.71	63.40	2.79	4.41
	[Low]	33.88	0.43	1.28	35.07	3.27	9.33

^1^ X: Mean. ^2^ SD = Standard deviation. ^3^ CV = Coefficient of variation.

**Table 2:** Recovery of SAA after mixing in different proportions two samples with high and low concentrations of serum amyloid A (SAA) with Fujifilm (method A), Eurolyser Diagnostica (method B) and Eiken (method C).

Method	% Sample High-[SAA]	% Sample Low-[SAA]	Recovery (%)
	100	0	
	90	10	109.91
A	50	50	112.12
	10	90	81.15
	0	100	
	100	0	
	90	10	101.26
B	50	50	117.86
	10	90	81.01
	0	100	
	100	0	
	90	10	107.10
C	50	50	112.48
	10	90	85.75
	0	100	

## Data Availability

Raw data can be obtained from the authors upon reasonable request.
